# Effects of Cold Air on Cardiovascular Disease Risk Factors in Rat

**DOI:** 10.3390/ijerph9072312

**Published:** 2012-06-29

**Authors:** Bin Luo, Shuyu Zhang, Shoucun Ma, Ji Zhou, Baojian Wang

**Affiliations:** 1 School of Applied Meteorology, Nanjing University of Information Science and Technology, 219 Ningliu Road, Nanjing 210044, China; Email: luob04@yahoo.com.cn (B.L.); msc_ever@163.com (S.M.); zhoujigood@163.com (J.Z.); 2 Key Laboratories of Arid Climatic Change and Reducing Disaster of Gansu Province, Lanzhou Institute of Arid Meteorology, China Meteorological Administration, 2070 Donggang East Road, Chengguan District, Lanzhou 730020, China; 3 Lanzhou Central Meteorological Observatory, 2070 Donggang East Road, Chengguan District, Lanzhou 730020, China; Email: baojianwang@126.com

**Keywords:** cold air exposure, CVD risk factors, myocardial injury indicators

## Abstract

The purpose of this study is to explore possible potential implications of cold air in cardiovascular disease (CVD) risk in rats. Healthy *Wistar* rats were exposed to artificial cold air under laboratory conditions, and their systolic blood pressure, heart rate, vasoconstriction, CVD risk factors, and myocardial damage indicators after cold air exposure were determined and evaluated. Systolic blood pressure, whole blood viscosity, and plasma level of norepinephrine, angiotensinⅡ, low density lipoprotein, total cholesterol, and fibrinogen in treatment groups increased significantly compared with control groups. No significant variations were found in plasma Mb and cTnT and myocardial tissue between the treatment and control groups. Results indicate that: (1) higher levels of SBP, WBV and LDL/HDL, total cholesterol (TC), and FG in blood may indicate higher CVD risks during cold air exposure; (2) cold air may exert continuous impacts on SBP and other CVD risk factors.

## 1. Introduction

Cold stress contributes to elevations in blood pressure. Studies report that a sudden exposure to the cold can increase the blood pressure of humans by 20 mmHg, especially when there exists cold induced pain at the same time [1,2]. A short period of persistent low temperature stress could also lead to blood pressure elevation in both human and animal subjects [3,4,5,6,7]. Several epidemiological studies have indicated that blood pressure is permanently elevated during the cold season [8]. Chronic cold stress, usually lasting for more than three weeks, could also cause cold-induced hypertension in rats [9,10]. These cold-induced blood pressure elevations have been proven to be related to the increase in norepinephrine (NE), epinephrine (EPI), and angiotensin II (ANGII) concentrations in the blood after cold stress [11,12,13]. As critical vasoconstrictors, NE, EPI, and ANG II are essential components of the sympathetic nervous system (SNS) and the rennin-angiotensin system (RAS), the activation of which could lead to elevations in blood pressure. Acute cold stress accelerates the heart rate (HR), whereas a long period of cold stress could slow it down or make no change at all [2,14,15,16,17]. To date, known studies mainly focus on the impact of constant low temperatures; the impacts brought about by the process of decreasing temperature in a natural cold air on HR remain unclear, particularly when the temperature changes from time to time. Thus, exploration of the effects of cold air on the cardiovascular system and its related mechanisms are essential to better understand and prevent adverse impacts brought about by cold air. 

Abundant epidemiological evidence has indicated that cold air may bring about increases in cardiovascular disease (CVD) events and even induce CVD deaths [18,19]. Such events may be connected to the impact of the cold on CVD risk factors, such as blood pressure, whole blood viscosity (WBV), blood fibrinogen (FG) and lipids, among others. The literature shows increases in blood pressure, plasma cholesterol, plasma fibrinogen, as well as red and white blood cells, during the winter or upon exposure to the cold [20,21,22,23,24]. This observation could be used to explain the increase in CVD events in cold weather. Based on this knowledge, we hypothesized that the temperature drop process during exposure to cold air could also increase CVD risk factors. Epidemiological studies suggest a delayed peak in CVD events after a cold air [25,26,27]. Thus, we aimed to explore variations in these risk factors at different time points during cold air exposure. We simulated the temperature change process of a typical cold air period and exposed rats to cold conditions to determine their cardiovascular response and its possible effects on CVD risk. The cold air data used in this study were collected from a period when human studies were carried out. However, since most people stayed home during the cold air the results obtained only represent the effects produced by abrupt cold exposure. This study aims to further explore the health effects of natural cold to provide dependable knowledge for future studies on populations who may experience cold air. 

## 2. Materials and Methods

### 2.1. Definition of Cold Air and Data Collection

A cold air commonly occurs in the winter and early spring. It is a period of time in which the ambient temperature drops significantly. In China, cold airs are graded through the range of drop in the daily minimum temperature over 24 h, 48 h, or 72 h. The one-day daily minimum temperature is the minimum temperature over 24 h from 06:00 of the previous day to 06:00 of the current day. Similarly, the range of drop in the daily minimum temperature at 24 h, 48 h, or 72 h is the range of drop in temperature from the previous day’s minimum temperature to another minimum temperature 24 h, 48 h, or 72 h later.

**Figure 1:**
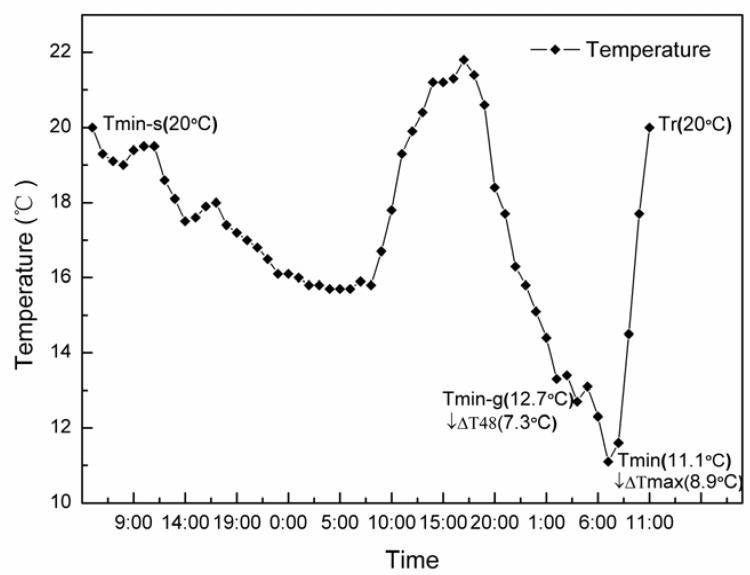
Temperature changes over time during the cold air process. Tmin-s: the starting minimum temperature of the cold air; Tmin-g: the minimum temperature for cold air grading; Tmin: the minimum temperature; Tr: the rewarming temperature; ↓∆T48 = Tmin-s − Tmin-g；↓∆Tmax = Tmin-s −Tmin.

The hourly temperature data of a cold air used in this study were collected from Zhangye City, a northwest city of China, in March 2011. This cold air was graded as a moderate cold air, with a drop in daily minimum temperature ranging from 6 °C to 8 °C according to the cold air grade standard of China. Details are shown in Figure 1. Over 7 year of collection of cold air observational data in Zhangye City (2004–2010), the range of cold air temperatures used in this study occurred about 103 times, or almost 80% of all cold air in the area; hence, it could be regarded as a frequently occurring cold air in Zhangye City.

### 2.2. Climate Simulator

In this study, a climate simulator (GDJS-500L, Pulingte Co., Tianjin, China) with a controllable temperature range of −20 °C to 120 °C was used to simulate the process of temperature change. The simulator can simulate not only the changeable temperature but also the constant temperature according to set values. The atmospheric pressure and humidity can also be controlled as needed. Data on temperature, relative humidity, and atmospheric pressure were automatically recorded every 10 s by an external computer. The rate of temperature drop was automatically modified along with the temperature interval between two temperature points. The animal keeping chamber of this climate simulator was an enclosed space of 80 cm × 80 cm × 80 cm, large enough to sustain experimental living conditions for the rats. The light in the chamber was controllable and provided a luminous flux similar to that under laboratory conditions. The oxygen concentration of the chamber was sustained at levels similar to those in the laboratory through a fixed air vent. After repeated testing, the climate simulator was considered well qualified to simulate temperature changes in this study.

### 2.3. Experimental Animals

Twenty-four healthy male *Wistar* rats, aged 10 weeks, weighing 305.1 g to 329.1 g, and with a systolic blood pressure of 116.5 mmHg to 119.3 mmHg, were obtained commercially from Vital River Laboratories (Beijing, China). All animals were kept in plastic and metal cages. Room temperature was sustained stably at 20 ± 2 °C, and a circadian rhythm of 12 h/12 h was maintained. All animals were given standard animal chow and water *ad libitum* and handled daily to minimize handling stress. Bedding was refreshed daily in every cage.

### 2.4. Experimental Protocol

All animals were kept in laboratory conditions for one week and then randomly divided into four groups with six animals in each:

Temperature drop group (TD): Exposed to the cold air temperature drop process in the climate chamber and until Tmin at 11.1 °C.

Temperature drop control group (TDC): Received no cold exposure and kept at room temperature (20 °C), served as control group for TD.

Entire temperature change group (TE): Exposed to the entire cold air process in the climate chamber until Tr at 20 °C

Entire temperature change control group (TEC): Received no cold exposure and kept at room temperature (20 °C), served as control group for TE.

Before cold exposure, all groups of animals were kept at a room temperature of 20 °C for one week as the control period. Then, TD and TE were moved into the chamber of the climate simulator for exposure to the simulated temperature change process of a cold air; TDC and TEC were kept in a room at a stable temperature of 20 °C and served as control groups. Relative humidity was controlled automatically at 45 ± 5 % in both thermal environments. All animals were given standard animal chow and water *ad libitum* throughout the entire experimental period. Padding was refreshed daily. Systolic blood pressure (SBP), HR, and body weight (BW) were determined. Determination of SBP and HR was carried out by the non-invasive tail-cuff method using an animal sphygmomanometer (BP-2006A, Softron, Beijing, China), which is widely used in many cold exposure experiments [6,10]. At Tmin = 11.1 °C, TD cold exposure was terminated, and the BW, SBP, HR and TDC were measured. After introduction of abdominal anesthesia with pentobarbital sodium (120 mg/kg i.p.), blood was collected through the abdominal aorta, and the heart was removed and fixed in 4% paraformaldehyde solution at pH 7.0. Three millilitres of blood was used for WBV determination; the rest was centrifuged at 3,000 rpm for 20 min to collect plasma, which was then kept at −80 °C until assay. When the temperature had returned to 20 °C, TE and TEC were subjected to the same procedure as TD. In all, the temperature dropping process persisted for 51 h and the cold air lasted for 55 h until the initial temperature was regained (20 °C). Actual times of the above procedures were conducted according to the time schedule in Figure 1. This research was conducted in accordance with the Declaration of Helsinki and with the Guide for Care and Use of Laboratory Animals as adopted and promulgated by the United National Institutes of Health. All experimental protocols were approved by the Review Committee for the Use of Human or Animal Subjects of Nanjing University of Information Science and Technology.

### 2.5. Assessment of Vasoconstrictors, CVD Risk Factors and Histological Changes

Plasma NE, EPI, endothin1 (ET1), ANG II, myoglobin (Mb),and cardiac troponin T (cTnT) were determined with an ELISA kit (Uscnlife, Wuhan, China); WBV was determined using a blood rheology system (LGR80, Steellex, Beijing, China) at shear rates of 10/s and 150/s. The Clauss method was used to measure the plasma concentration of FG (agents produced by Siemens Healthcare Diagnostic Products Gmbh, Marburg, Germany) [28]. Methods of GPO-PAP, CAT, and CHOD-PAP determination were applied to obtain the triglyceride (TG), low density lipoprotein (LDL), and high density lipoprotein (HDL) levels and total cholesterol (TC) [29,30,31,32,33] (agents produced by Sichuan Maker Biotechnology Co., Ltd., Chengdu, China). Histopathological changes in the heart were determined under an optical microscope (Olympus cx31) after paraffin embedding and hematoxylin-eosin staining.

### 2.6. Statistical Analysis

All results were analyzed with SPSS13.0. All of the results are shown as mean (SE, standard error). Means between different treatment groups were compared by Independent-sample T test. A 95% confidence limit was employed to explore significance.

## 3. Results

### 3.1. HR, BW, and SBP

**Figure 2:**
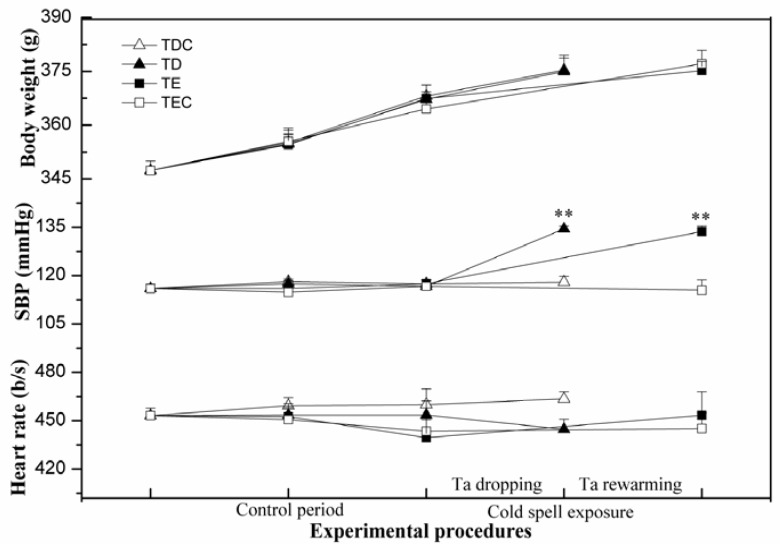
Variation in body weight, SBP, and HR of rats under controlled and cold air exposure periods. Data shown as mean + SE; n = 6 in each group. Compared with the control group,^**^ *P* < 0.005.

Throughout the entire period of controlled housing and cold exposure, all groups of rats showed a stable growth rate and no significant difference was found among them; no significant difference was observed either among the different groups in their HR. This suggests that the temperature dropping process of the cold air used in this study has no impact on the growth and heart rates. However, compared with the control groups, a marked increase in SBP was found in TD and TE after cold air exposure (*P* < 0.005); no significant difference was observed between TDC and TEC (Figure 2).

### 3.2. Vasoconstrictors: NE, EPI, ET-1, and ANGII

**Figure 3:**
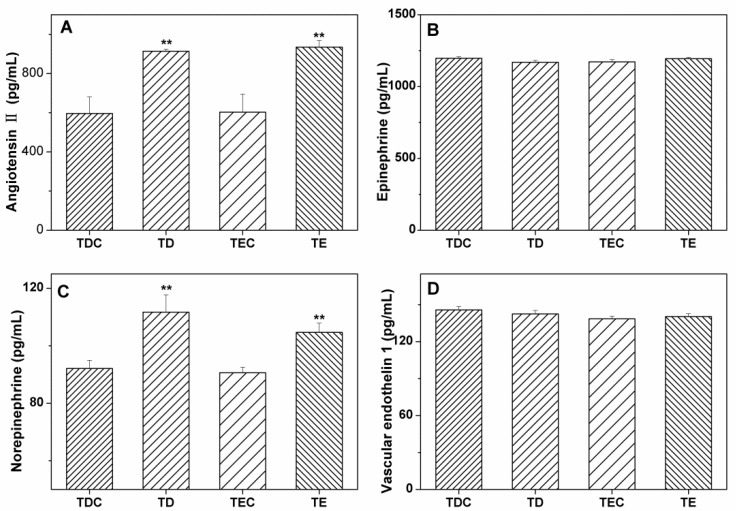
Effect of the temperature dropping process on plasma ANG II (A), EPI (B), NE (C), and ET1 (D) in *Wistar* rats. Results shown as a mean + SE, n = 6 in each group. Compared with the control group, ^**^ *P*< 0.005.

As shown in Figure 3, the temperature dropping process did not produce a significant effect on plasma concentrations of EPI and ET-1 in rats (*P* < 0.005), but it did induce NE to increase noticeably in both TD and TE (*P* < 0.005). 

No difference was found between TD and TE (*P* > 0.05); this finding indicates the continuous effect of cold stress on NE. The same results were observed for ANG II, suggesting the impact of the temperature dropping process of cold air exposure on RAS. 

### 3.3. CVD Risk Factors: WBV, FG, and Blood Lipids (Table 1)

At shear rates of 10/s and 150/s, both TD and TE displayed a higher WBV than their control groups (*P* < 0.005, *P* < 0.05), but no significant difference was found between them. TD had a higher concentration of plasma FG than TDC and TE (*P* < 0.005, *P* < 0.05), even if TE had a higher concentration of FG than TEC (*P* < 0.005). Among the blood lipids results, including TG, TC, LDL, and HDL, the temperature change process of the cold air mainly induced plasma TC and LDL to increase (*P* < 0.005) and TG to decrease (*P* < 0.005); no effects on the plasma HDL, both in TD and TE, compared with control groups were observed (*P* > 0.05). TD had a higher concentration of plasma TC and LDL than TE (*P* < 0.05), indicating slight recovery after the temperature rewarming process. In contrast with the control groups, the LDL/HDL was found to be higher both in TD and TE (*P* < 0.005). 

**Table 1:** Effects of the temperature dropping process on WBV, FG, and blood lipids (mean (SE), n = 6 in each group)

CVD risk factors		TDC	TD	TEC	TE
**Whole blood**	**10/s**	10.51(0.72)	16.34 (2.03) ^**^	12.23(0.55)	17.13(0.91) ^*^
**Viscosity (mpa.s)**	**150/s**	4.45(0.27)	11.11(0.23) ^**^	4.87(0.16)	6.08(0.47)^*^
**Fibrinogen (g/L)**		1.85(0.04)	2.29(0.05) ^**^	1.79(0.03)	2.07(0.07) ^**,†^
**Blood lipids (mmol/L) **	**TC**	1.26(0.04)	2.27(0.12) ^**^	1.29(0.07)	1.70(0.09) ^**, ††^
	**TG**	0.51(0.03)	0.27 (0.02) ^**^	0.43(0.06)	0.26(0.03)
	**HDL**	0.71(0.03)	0.83(0.04)	0.77 (0.03)	0.69(0.05) ^†^
	**LDL**	0.58(0.06)	1.47(0.13) ^**^	0.53(0.07)	1.02(0.08) ^**,††^
**LDL/HDL**		0.84(0.11)	1.76(0.12) ^**^	0.69(0.08)	1.51(0.16) ^**^

Compared with the control group, ^*^ *P* < 0.05, ^**^ *P* < 0.005; Compared with the **TD** group, ^†^ *P* < 0.05, ^††^ *P* < 0.005. **TC**—total cholesterol; **TG**—triglyceride; **LDL**—low density lipoprotein; **LDL**—high density lipoprotein.

### 3.4. Myocardial Damage Indicators: Mb, cTnT, and Histopathological Changes in the Heart

After statistical analysis, no significant difference in plasma Mb and cTnT was found between any group (*P* < 0.05, Table 2). 

**Table 2:** Results of plasma Mb and cTnT levels and cases of hearts with lesions. (mean (SE), n = 6 in each group).

Myocardial injury markers	TDC	TD	TEC	TE
**Cardic Troponin T (pg/mL)**	9.38(0.79)	8.40(0.34)	9.29(0.32)	8.65(0.30)
**Myoglobin (pg/mL)**	323.68(11.67)	356.75(16.33)	323.88(18.17)	368.46(19.14)
**Focal Myocardial Lesions**	-	-	-	-

TDC: temperature drop control group; TD: temperature drop group; TEC: entire temperature change control group; TE: entire temperature change group.

**Figure 4:**
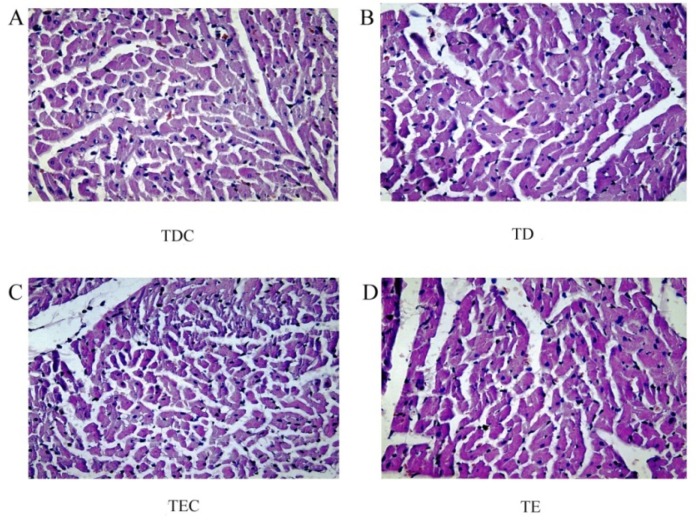
Left heart ventricle of sample rats from TDC, TD, TEC, and TE; n = 6 in each group. Hematoxylin-eosin 400.

Under optical microscopic observation, no sign of possible damage to the heart, necrosis, atrophy of myocardial fiber cells, or inflammatory cell infiltration, among others, was observed (Figure 4).

## 4. Discussion

During the entire cold air process, the temperature dropped for about 8.9 °C within 51 h, with an average drop rate of 0.17 °C/h. This slow temperature change rate may have induced adaptation of the rats to the environmental conditions to some degree so as not to produce any impact on their HR. The HR findings may also be explained by the unchanged plasma level of EPI through the entire temperature change process, as it often acts as a positive regulator of HR. However, the cold air induced SBP to increase, not only in TD but also in TE. This finding may be explained by the increased plasma level of NE and ANG II in TD and TE. As critical components of SNS and RAS, increased plasma NE and ANG II could contribute to vasoconstriction and lead to the elevation of SBP. Therefore, the cold air may have activated SNS and RAS and induced SBP elevation in the rats, similar to the mechanism by which constant low temperature stresses induce SBP elevation [17]. 

As an easily observable and sensitive index of the cardiovascular system towards the cold, SBP has been reported to be more sensitive than the diastolic pressure in predicating the occurrence of CVD events [34,35]. Constantly elevated blood pressure could increase myocardial load and oxygen exhaustion and reduce blood flow to the brain, which could eventually induce myocardial and cerebral infarctions [36,37]. Among CVD deaths caused by the cold, many of them are characterized by a history of hypertension [38,39,40], so SBP elevation may be a critical contributing factor. Cardiovascular damage caused by constantly elevated BP has already been indicated in cold-exposed animals (with constant low temperatures) [9,41]. Aside from SBP elevation, the increase in plasma ANG II could also be a critical contributing factor to the higher occurrence of CVD events during cold weather, because its increase can lead to inflammatory response vessels and induce vascular endothelium dysfunction [42]. Some traditional CVD risk factors, such as WBV, plasma FG, TC, and LDL [43,44,45,46,47,48,49] were also found to be higher in treatment groups than in control groups. The increase in WBV may indicate increases in some coagulation factors, such as red cells, FG, blood lipids, platelet, and haemoglobin [23,50,51]. Therefore, the increase in WBV may increase the risk of thrombosis in coronary and cerebral arteries. In addition to being an indispensable coagulation factor, increased plasma FG could also directly join the process of atherosclerosis by binding fibrin and its degradation products-FDP-to induce pro-inflammatory responses [51,52]. The CVD risk contributed by the increased plasma level of TC and LDL is also closely related to their function to induce atherosclerosis by inducing manifestation of adhesion molecules and vascular endothelium dysfunction [53,54]. In addition, the CVD risk predictor-LDL/HDL-was also significantly higher in both TD and TE than the controls. A study reported that the LDL/HDL ratio is an excellent predictor of CVD risk, being more accurate than LDL or HDL alone [55]. Therefore, the increase in CVD risk factors and predictors in rats after cold exposure suggests that a cold air may increase CVD risk. Moreover, the results yielding significantly higher levels of CVD risk factors, such as SBP, WBV, plasma FG, TC and LDL, and a higher CVD risk predictor of LDL/HDL in the TE group may also indicate higher CVD risks even after Ta has recovered. CVD risk induced by WBV, FG, TG, and LDL is a latent process; higher levels of CVD risk factors in TD do not mean that the condition poses a unique and higher CVD risk than TE. 

Sun *et al.* reported that chronic cold exposure induced cardiac hypertrophy, even when the blood pressure elevation had been depressed [56]. However, in our study, the temperature change process of the cold air did not cause any damage to the heart, as proven by the unchanged plasma levels of myocardium damage indicators—Mb and cTnT [57,58,59] and the unaltered myocardial tissue under the microscope. The short period of cold stress in this study may be the very reason for this finding, even if it induces elevation in the blood pressure in rats. Hence, moderate cold air does not induce heart damage in healthy rats. 

In this study, a natural cold air was successfully simulated and applied as a cold stress. The results indicate a mechanism by which the cold air may also increase blood pressure by activating SNS and RAS, similar to studies which obtained elevated blood pressure by giving fixed lower temperature stress to animals. Since rats are able to maintain their core temperatures constant during exposure to cold 5 ± 2 °C [41,60], the increase in SBP, WBV, FG, TC and LDL and decrease in TG in the blood may be due to their adaptation to the cold. Another probable explanation for these increases may be the dieresis induced hemoconcentration during cold exposure, suggested in other studies [61]. However, we did not study the dieresis of cold, but we did observe soppier padding in treatment groups than control groups. The increase in CVD risk factors may not be related to any cardiovascular system damage in healthy rats, but they may lead to cardiovascular system damage in CVD subjects. Since a delayed phenomenon after Tmin was found in the peak occurrences of CVD events, the significantly increased risk factors in the temperature rewarming group may be regarded as a risk sign for the progress of atherosclerosis. The difficulty here is that the time by which this phenomenon was observed was limited to a few hours. Although most people stay indoors when a cold air comes, some people, especially those living in rural areas, with no heating measures or who have jobs outdoors, cannot avoid exposure to a cold air. Therefore, this study is necessary to unveil the cardiovascular risks that a cold air may bring. Rats were used as research subjects in this study, but they may not completely simulate the condition of a whole human body being exposed to a cold air. Therefore, further human studies are necessary to prove these obtained results. Even if rats are able to maintain their core temperatures constantly during cold exposure 5 ± 2 °C [41,60], the specific variation in the core temperature during this cold air was unknown because of the limitations of the study; thus further research is needed.

## 5. Conclusions

In conclusion: (1) higher levels of SBP, WBV and LDL/HDL, total cholesterol (TC), and FG in blood may indicate higher CVD risks during cold air exposure; (2) cold airs may exert continuous impacts on SBP and other CVD risk factors.
